# Co-constructing stories with patients with heart failure to envision future remote care possibilities: a qualitative interview and sketching study

**DOI:** 10.3389/fdgth.2026.1894748

**Published:** 2026-09-03

**Authors:** Wessel W. Nieuwenhuys, Hannah C. van Iterson, Jeroen A. A. van de Pol, Mayke M. C. J. van Leunen, Ignace L. J. de Lathauwer, Mathias Funk, Rong-Hao Liang, Hareld M. C. Kemps

**Affiliations:** 1 Department of Industrial Design, Eindhoven University of Technology, Eindhoven, Netherlands; 2 Netherlands Heart Network, Eindhoven, Netherlands; 3 Eindhoven MedTech Innovation Centre (e/MTIC), Eindhoven, Netherlands; 4 Department of Electrical Engineering, Eindhoven University of Technology, Eindhoven, Netherlands; 5 Department of Cardiology, Máxima Medical Centre, Veldhoven, Netherlands

**Keywords:** co-constructing stories, heart failure, participatory design study, patient-centered design, patient-centered care, remote patient monitoring (RPM)

## Abstract

**Introduction:**

Although remote patient management (RPM) shows promise for improving chronic heart failure (HF) management, its effectiveness depends on technology acceptance, usability, and patient engagement. However, patients are often involved only after systems are implemented, limiting opportunities to align RPM with their needs. This study explored patients' expectations, wishes, and concerns regarding future RPM using participatory design methods to inform more patient-centred RPM.

**Methods:**

Patients with HF using RPM were recruited from a Dutch hospital for individual, semi-structured interviews. We employed a co-constructing stories approach, inviting participants to imagine future scenarios of remote care. Guided by narrative prompts about potential developments in RPM, participants were encouraged to discuss how such technologies might shape their experiences. During the interviews, one researcher concurrently created sketches of the participants' answers, creating an evolving trace of the dialogue. The visual data were subsequently analysed using Annotated Visual Analysis to identify themes.

**Results:**

Six patients were interviewed, producing 7 h of audio and 18 A3 pages of sketch notes. Four key themes emerged: *Data Feedback,* reflecting patients' desire to receive clear and constructive feedback that supports self-management beyond alert-based monitoring, to be able to take more responsibility for their health through RPM; *Invasiveness,* including camera-based monitoring or collection of non-medical data, was perceived as a barrier to adoption, although concerns were reduced when monitoring was clearly explained and considered proportional to disease severity; *Adaptability and Integration* highlighted the need for RPM to extend support beyond monitoring and to remain useful in daily life; *Healthcare Provider contact* emphasised that human interaction is irreplaceable, with empathy from healthcare professionals fostering trust in the system and patients' confidence in managing their health.

**Conclusion:**

Inviting patients to reflect on remote care provided valuable insights into their needs and expectations for future RPM. Participatory design methodologies facilitated rich discussions about patients' experiences, needs, and expectations regarding future RPM. Patients emphasised RPM should be actionable, minimally invasive, adaptable to daily life, and complemented by human contact. This highlights the importance of socio-technical systems that empower self-management while maintaining professional support. These findings can guide more human-centred RPM designs for HF care.

## Graphical Abstract

**Figure:**
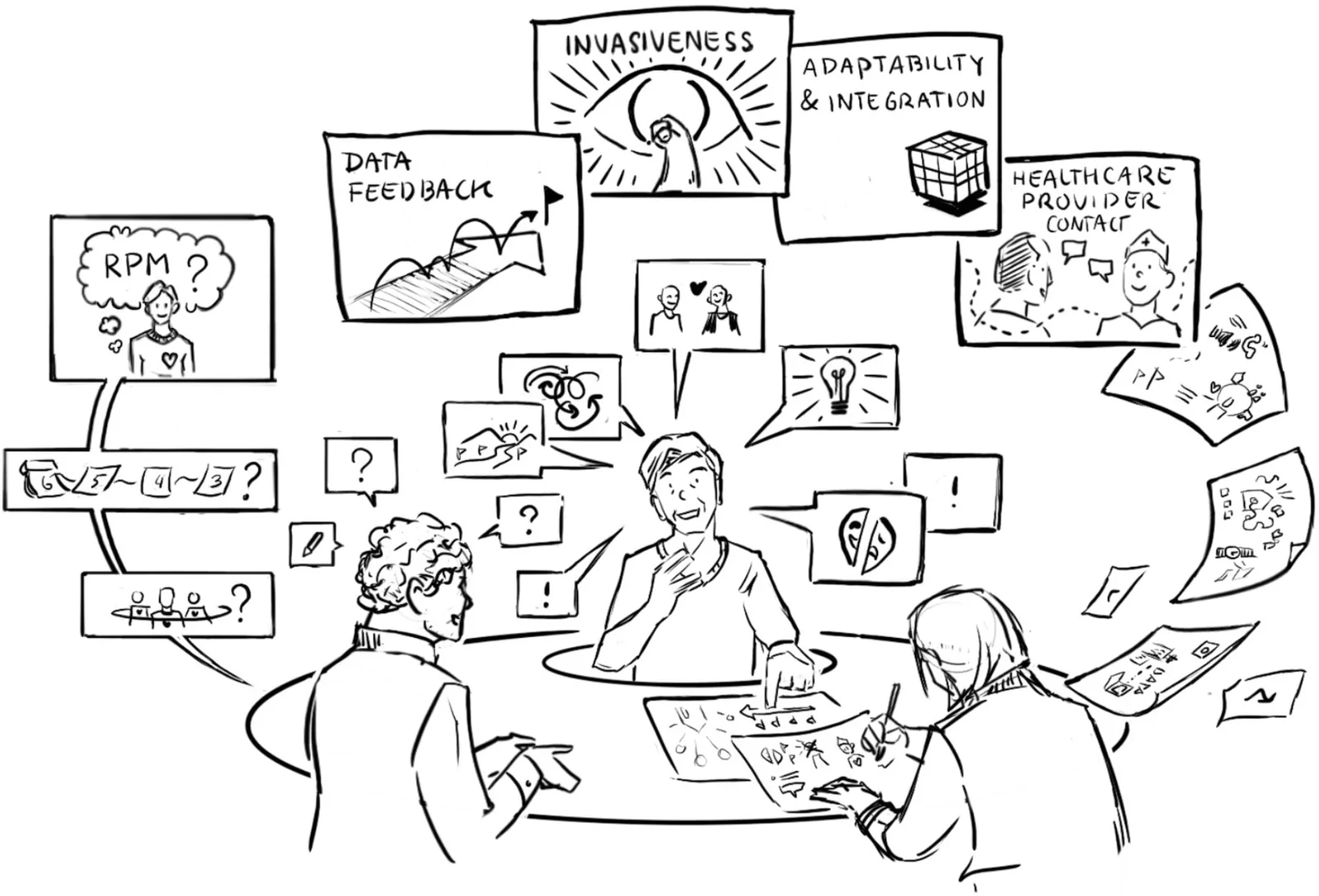
Impression of the co-constructing stories method employed during the interviews. Participants’ responses are interpreted through sketches, providing an immediate opportunity for reflection and a shared reference point during the discussion.

## Introduction

Remote Patient Management (RPM) is a form of hybrid care that allows clinicians to monitor and manage their patient's chronic or acute medical conditions. Patients are provided with disease-related sensors and communication platforms, which they can use to measure and digitally communicate their physiological data to their respective healthcare providers (HCPs) (1). It has shown to have promising effect on the clinical outcomes and reduce the use of acute care, even when using non-invasive technologies only (2–4). Furthermore, patients report high rate of satisfaction, and equal quality of life (5). This growing evidence base has supported the expansion of RPM into the management of a wide range of chronic conditions.

One chronic condition for which RPM has become increasingly relevant is heart failure (HF), a condition characterised by the need for continuous medical management to prevent recurrent acute exacerbations and subsequent (re-)hospitalisation (6). Currently, RPM for HF is typically delivered through mobile or web-based applications in which patients manually enter self-measured physiological data. These data are then shared with HCPs and subsequently reviewed asynchronously.

In recent years, research has focused on optimising RPM workflows, improving clinical outcomes, and reducing patient and HCP burden (7, 8). However, like with many healthcare innovations, clinical effectiveness and technical feasibility often take precedence over patients' lived experiences, values, and concerns (9, 10). As a result, interventions may not achieve their intended effectiveness, due to suboptimal patient adherence and acceptance (11–13). Patients are often involved too late in the development of medical innovations, requiring manufacturers to retrofit solutions to meet their needs to ensure uptake. By engaging patients early in the design process, the patient-facing side of healthcare technologies can be better tailored to real-world needs, thereby improving adoption and adherence (14, 15). Participatory design offers a structured approach to achieving this, by actively involving end-users, such as patients, throughout the early stages of development. As a result, it has become a central approach in human–computer interaction research over the past decade (16–19).

One of the methods employed in participatory design studies is co-constructing stories (20). Building on participant's retrospective experiences, this narrative-based approach enables stakeholders to collaboratively build prospective narratives about potential future scenarios to collect in-depth feedback on design concepts, without the need for physical, working prototypes. Through non-directive questions and prompts that draw on both past experiences and future possibilities, participants can express hopes, concerns, latent desires, and tacit needs, as well as functional, emotional, and ethical considerations such as privacy, autonomy, and trust. Participants begin by reflecting on what feels familiar, helpful, or challenging in their current daily routines. They are then introduced to a fictional design concept that invites them to imagine how their daily life might change if such a concept were part of their care. By rooting their expectations of the future in their current lived experiences, co-constructing stories can not only elicit patients' explicit preferences but also uncovers needs and wants that might otherwise remain unsaid (20). Applying this methodology in the context of remote care can therefore provide a rich, patient-centred foundation for the future design of RPM.

## Objective

By using the co-constructing stories method, this participatory design study aims to elicit reflective conversations about how RPM for patients with HF should, or should not, be designed to be in line with patients' preferences with regards to monitoring. Insights gained can then inform the development of patient-facing aspects of RPM, ensuring they are tailored to users' needs and wishes.

## Methods

### Participants

Participants were recruited from the Máxima Medical Centre, a non-university teaching hospital in the Netherlands that has implemented RPM for patients with HF. Eligibility for enrolment in the hospital's HF RPM program was based on the following criteria: (1) a diagnosis of chronic HF, irrespective of aetiology and left ventricular ejection fraction (LVEF), established during either hospitalisation or an outpatient consultation; (2) a clinical status considered sufficiently unstable that the patient's HCP saw benefit in remote management (i.e., recent hospital admission for acute decompensated HF or fluctuations in fluid status requiring changes in diuretic medication); (3) sufficient digital literacy, either by the patient or with the support of a family member; and (4) proficiency in the Dutch language. Patients are asked to monitor 5 times a week in the early, unstable, phase of HF right after diagnosis, and once HF stabilises and becomes chronic, 1 time per week.

During each monitoring session, patients measured their body weight using a provided, or already self-owned, digital scale and their blood pressure and heart rate using a provided blood pressure monitor. They subsequently manually entered these measurements, together with responses to a short questionnaire on heart failure-related symptoms, into the Luscii mobile application (Luscii Healthtech B.V., the Netherlands). The data were securely transmitted to the hospital, where they were reviewed by the responsible HCP. When deemed necessary, the HCP initiated appropriate follow-up actions, such as scheduling a consultation or adjusting the patient's medication. These communications were conducted by phone. Luscii is a CE-marked Class IIa medical device certified under the European Medical Device Regulation (MDR).

HCPs purposively identified adult (≥18 years) patients with HF using RPM who, based on their clinical experience with these patients, were considered able to reflect on and articulate their perspectives on RPM. Patient selection considered characteristics such as adherence, experience with RPM, and previous consent to be contacted for future research following participation in a prior study. Those expressing interest were subsequently contacted by the research team for participation in the interviews. Patients were contacted by phone and interviewed sequentially, with ongoing assessment of emerging themes. Recruitment continued until the research team determined that additional interviews were unlikely to provide substantial new insights relevant to the research question.

The Medical Ethics Committee of Máxima Medical Centre (the Netherlands) determined that the study did not fall under scope of the Medical Research Involving Human Subjects Act (WMO) and therefore did not require further approval. (METC-code: N24.039).

### Study design

In this participatory design study, we invited nine patients with HF to participate in semi-structured interviews using the co-constructing stories approach. Of these, six patients agreed to participate, allowing us to explore how patients with HF currently experience RPM and how they envision its future role in their at-home care.

During each interview two researchers were present. One researcher (interviewer, WWN) was focussed on guiding the conversation, while the other researcher (sketcher, HCvI) performed the task of concurrently visualising the participants answers by sketching. WWN is a Dutch male PhD candidate researching RPM for HF with a Biomedical Engineering background. HCvI is a Dutch female PhD candidate and design researcher in co-design for healthcare. Neither researcher had a prior relationship with participants. Participants were informed about the researchers' backgrounds, the study purpose, and their role in exploring future perspectives on RPM.

Each interview was conducted individually, at a location most convenient for the participant, with options including the hospital (Máxima Medical Center), university (Eindhoven University of Technology), or the participant's home. The interviews took approximately 1 h and were conducted in Dutch. An impression of the interview setting and the role of sketching has been depicted in the Graphical Abstract.

#### Sketching as a reflective tool during interviews

During the semi-structured interviews, the sketcher concurrently visualised the participants' answers as they were given through sketching, with the participants encouraged to actively react. By physicalising participants' responses as they emerged, sketching created a visible trace of dialogue that helped reveal reasoning, supported recall, and invited moments of reflection (21–23). This direct visual feedback enabled the participant, interviewer, and sketcher to find common ground and develop deeper reflections on needs and wants for future designs. Visual representation thus helped externalise ideas in complementary ways, allowing both researchers and participant to actively confirm that they were referring to the same concepts (24).

In the co-constructing stories methodology, participants typically sketch their own stories or add to sketch templates provided by researchers while describing their experiences and expectations (20). In this study, we adapted this approach by having the researcher sketch the participants' stories, as participants showed reluctance to create sketches themselves. To ensure that the researcher's visual interpretation remained aligned with the participants' stories, participants were invited to actively engage with the sketches throughout the process by adding their own drawings or commenting on and modifying those created by the sketcher. To further foster this engagement, the sketcher periodically invited the participant to actively reflect on the visual interpretations. Different colours were used to distinguish between the sketcher's original sketches and later additions made due to reflections of participants. Ultimately, the final visual output was reviewed by the participant to ensure it accurately reflected their input.

#### Interview structure and research team

The interviews were divided into three main sections. The full, translated, prompts for the co-constructing stories sections (Section 1, Section 3) and the questions of the semi-structured interviews (Section 2) are made available in Supplementary Appendix A. The guiding questions for Section 2, together with the prompts and guiding questions for Sections 1 and 3, were read to participants verbatim. Follow-up questions were then guided by participants' responses, allowing them to shape the direction of the discussion.

##### Section 1—warm-up scenario

In the first section, participants were accustomed to the questioning approach of the co-constructing stories methodology using a warmup scenario. The participants were prompted with a playful scenario in which they were asked to envision a challenge with their family where their sitting time throughout the day was monitored using sensor-equipped blankets on sitting surfaces. Participants were asked to imagine waking up on the first day of the challenge, having these sitting blankets placed throughout their home, and to reflect on the feelings this evoked and its influence on their usual behaviour.

##### Section 2—current experiences

In the second part of the interview, participants were asked to describe their current experiences with RPM for HF. The aim of this section was to gain insight into how RPM is currently integrated into participants' daily lives and to assess how it impacts these lives. This section followed a semi-structured interview format. Answers collected in this section of the interview were further used in the third section to ground the participants' answers on the potential future in current lived experiences.

##### Section 3—future scenario

In the third section, participants were presented with a scenario involving an advanced, largely automated home-monitoring system. This system was described as requiring only minimal patient input, while providing continuous monitoring of both clinical parameters and contextual information related to lifestyle. It was further described as capable of showing patients information regarding the collected data. Repeating the procedure of the first section, participants were asked to imagine their first day living with such a system in their home. This discussion served as an entry point for exploring participants' hopes, concerns, and expectations regarding future RPM technologies. Using the participants responses, follow-up questions were asked. Participants were encouraged to modify and reshape the imagined home-monitoring system throughout the interview, allowing the discussion to evolve according to their own ideas and expectations rather than remaining constrained by the initial scenario. Based on participants' answers and anecdotes, alternative scenarios were introduced in later parts of the interview to deepen discussion and reflection. These scenarios included having family over, days on which measured values indicated possible issues, and days involving many outside activities.

### Data collection and analysis

The outputs from the interview sessions included researcher-produced sketches, with immediate additions based on the participants' reflections, as well as audio recordings and automated transcriptions of the full interviews. The automated transcriptions were reviewed and edited by one of the researchers for accuracy. The sketches served as the primary source of results, while the transcriptions were used when necessary to provide extra context to the researcher while analysing the sketches. By referring to the transcriptions when uncertainties arose during analysis, we sought to minimise the risk of losing information.

The two researchers independently analysed the sketches to interpret the content, make annotations, and identify emerging themes, using the AVA (Annotated Visual Analysis) method. AVA primarily relies on static visual data, such as sketches, as the basis for analysis.

First, through the placement of annotations, researchers separately reflected on the visual data, and then grouped these annotations, identifying themes within participants' responses (25). Themes were allowed to emerge inductively through engagement with the sketches to ensure that insights arose naturally from the data. Subsequent discussions between the researchers were held to reach consensus on the identified themes. If no consensus was reached, a third researcher was consulted. Within the identified themes, the researchers collaboratively grouped related annotations into overarching messages that captured the central insights emerging from participants' answers. The online whiteboard software Miro was used throughout the analysis process, providing a structured record of the progression from initial annotations to final themes and overarching messages (26).

The analysis aimed to explore the lived experiences of patients with HF using RPM and their future needs, identifying connections and overlaps between themes to define design requirements for future RPM implementations.

**Figure 1:**
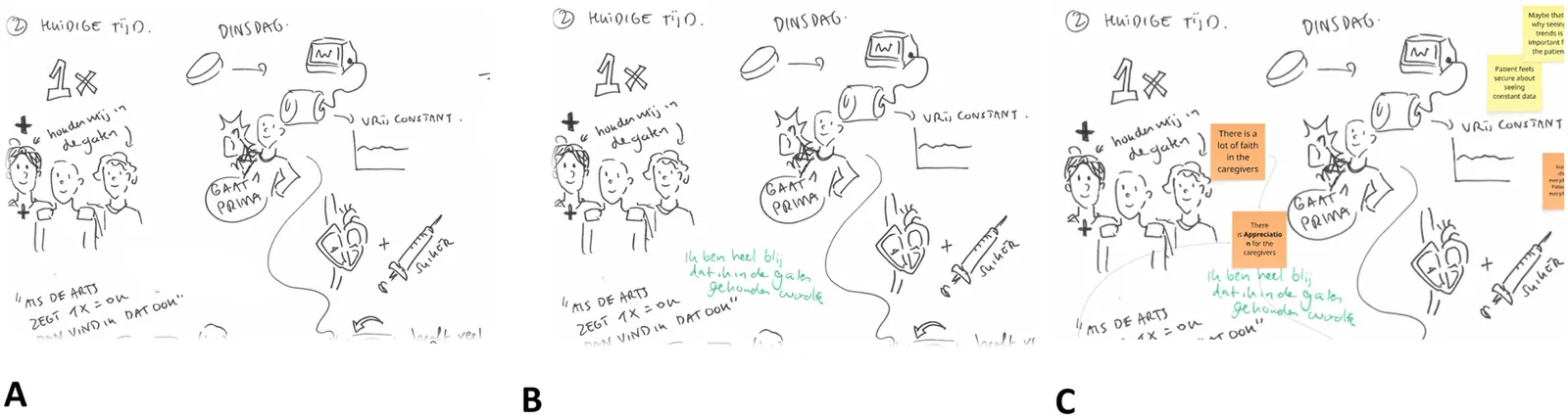
Sketching and AVA result extraction. (**A**) Original researcher made sketch. Generated by the sketching researcher during the interview with the participant, based on their interpretation of the participants answers. (**B**) Additional information added in a different colour based on the participants reflection after the sketcher showed their initial sketch to the participant. (**C**) Sketches annotated with identified themes by the researchers using the AVA methodology. Different colours were used to easily distinguish between the themes. Generated after the interview, during data analysis.

Figure 1 provides an overview of the process from the initial sketch to the researcher-annotated and thematically coded version.

## Results

### Number of people included

A total of 6 participants were interviewed (4 male, 2 female, mean age 63.7 ± 10.9 years). Of these participants 5 opted for an interview at their home, while the last opted for an interview at the hospital. Five participants were in the “stable” phase of monitoring at the time of the interview, measuring once per week, while one participant was earlier in the monitoring phase, i.e., the “unstable” phase, measuring five times per week. All five stable participants were initially enrolled in, and thus had experience with, the unstable monitoring phase (Table 1). In total, the interviews generated 18 A3 pages of sketches and 7 h and 14 min of audio material for analysis.

**Table 1:** Patient characteristics.

Participant number	Gender	Age	Phase of monitoring
1	Male	43	Stable (1x/week)
2	Female	62	Stable (1x/week)
3	Male	73	Stable (1x/week)
4	Male	77	Stable (1x/week)
5	Female	66	Stable (1x/week)
6	Male	61	Unstable (5x/week)

### Identified themes

Using the AVA method to analyse the researcher-generated sketches led to the identification of four main themes: Adaptability and integration, reflecting the impact of HF on patients' daily lives and the consequent need to integrate RPM into their routines; preferences regarding the type and delivery of feedback based on participant data; the perceived invasiveness of RPM in daily life; and the importance of maintaining HCP contact even when monitoring from home.

#### Adaptability and integration of remote patient management in daily life with heart failure

**Figure 2:**
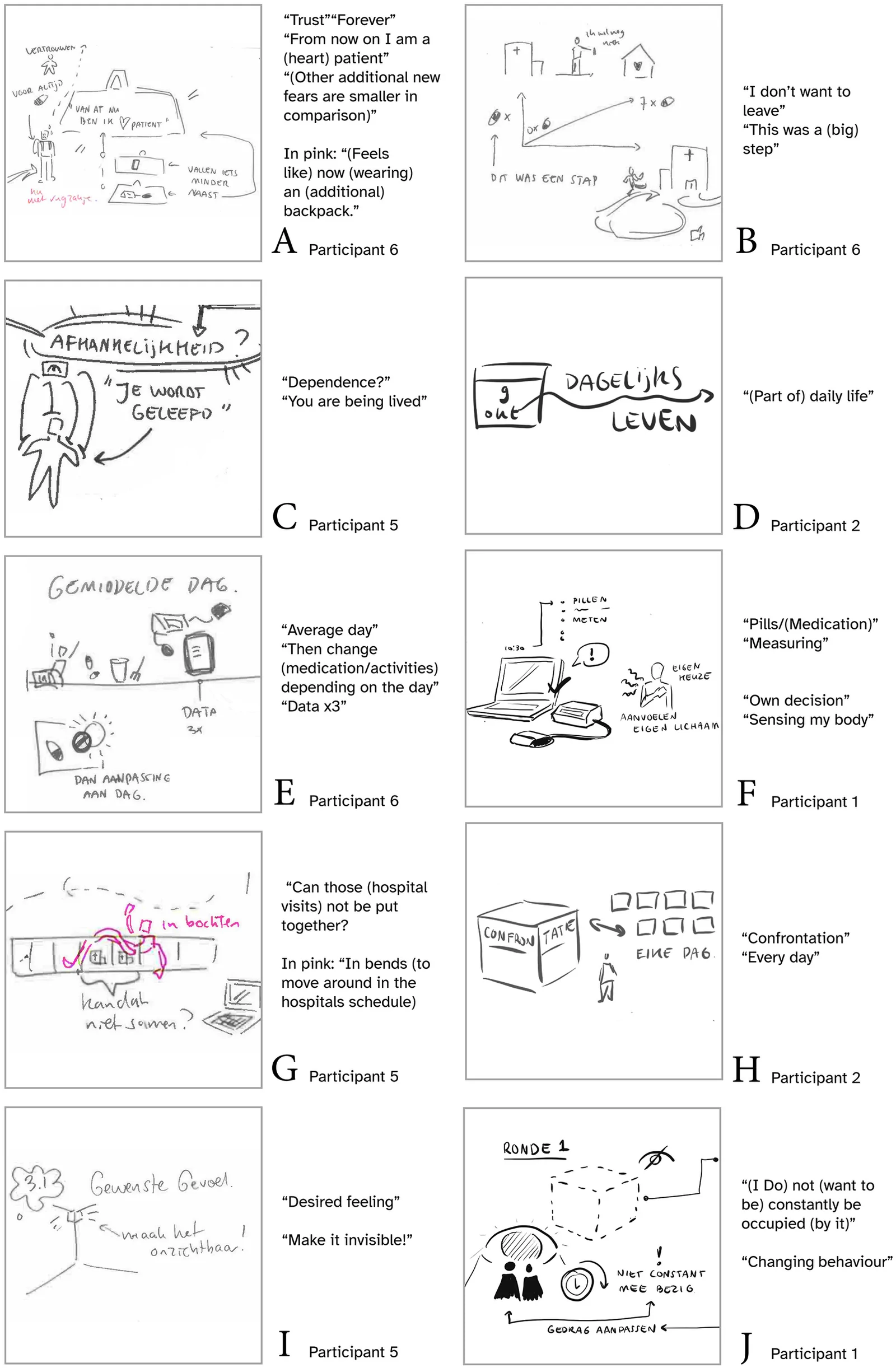

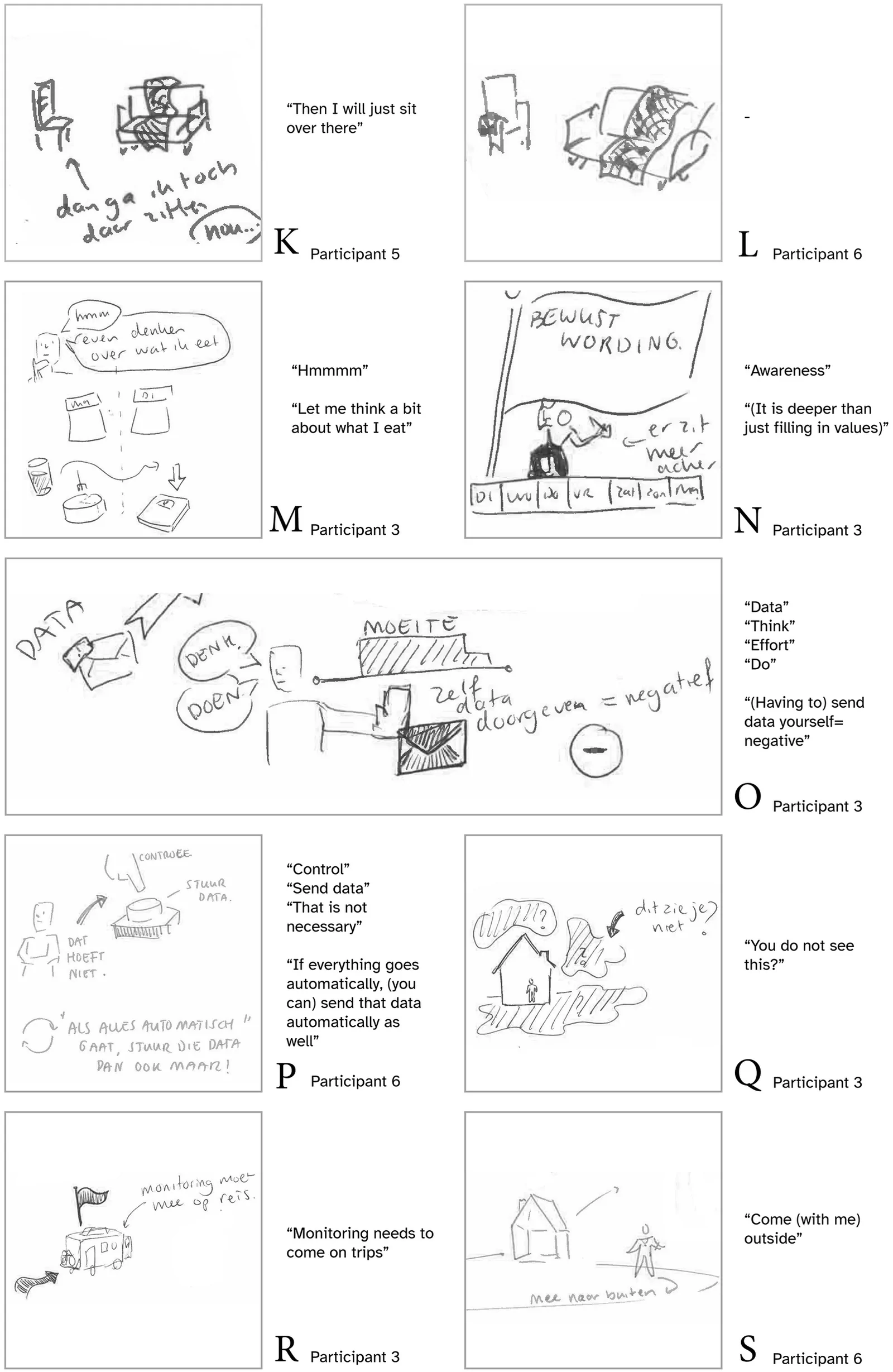
Researcher generated sketches, supplemented with participants' reflections shown in a different color. English translations are provided in the final column and are presented from top left to bottom right. Translations shown in brackets indicate additions made to improve clarity in English. **(A)** Researcher-generated sketch of an answer given by participant 6, depicting the impact heart failure has on their self-perception. **(B)** Researcher-generated sketch of an answer given by participant 6, depicting their unwillingness to leave the hospital after initial admission. **(C)** Researcher-generated sketch of an answer given by participant 5, depicting the participant's uncertainty about living with HF. **(D)** Researcher-generated sketch of an answer given by participant 2, depicting the integration of RPM into the participant's daily life over time. **(E)** Researcher-generated sketch of an answer given by participant 6, depicting how RPM started to fit in their daily routines. **(F)** Researcher-generated sketch of an answer given by participant 1, depicting the patients interaction with the RPM system, based on how well they are feeling that day. **(G)** Researcher-generated sketch of an answer given by participant 5, depicting how RPM helped reduce the amount of hospital visits that the participant usually had difficulty planning their day around. **(H)** Researcher-generated sketch of an answer given by participant 2, depicting the continuous reminder that RPM gave them of their HF. **(I)** Researcher-generated sketch of an answer given by participant 5, depicting the wished-for unobtrusiveness of the system. **(J)** Researcher-generated sketch of an answer given by participant 1, depicting the wished-for unobtrusiveness of the system. **(K)** Researcher-generated sketch of an answer given by participant 5, depicting the participant's ideas on how to cheat the monitoring system. **(L)** Researcher-generated sketch of an answer given by participant 6, depicting the measuring blankets from the first phase of the interview, and how the participant could cheat this system. **(M)** Researcher-generated sketch of an answer given by participant 3, depicting the participant's awareness of monitoring days, and how that impacts their health-related behavior. **(N)** Researcher-generated sketch of an answer given by participant 3, depicting the participant's realisation that the created awareness by monitoring could be seen as beneficial. **(O)** Researcher-generated sketch of an answer given by participant 3, depicting how RPM that requires a lot of input from a patient will take effort, and give a negative experience. **(P)** Researcher-generated sketch of an answer given by participant 6, depicting the integration of RPM into the participant's daily life over time. **(Q)** Researcher-generated sketch of an answer given by participant 3, depicting the participant's confusion about how the RPM system currently would only monitor inside their homes. **(R)** Researcher-generated sketch of an answer given by participant 3, depicting the participant's wish to be able to take their RPM system on vacation. **(S)** Researcher-generated sketch of an answer given by participant 3, depicting the participant's wish to be able to take their RPM system outside of their homes.

Receiving a diagnosis of HF was described as an impactful moment in the lives of the participants. The diagnosis not only required them to adapt to new living rules but also marked a change in how they perceived themselves, as they came to see themselves as a person with HF. This was associated with feelings of fear, uncertainty, and concern about what the diagnosis would mean for their future (Figure 2A). Participants described the hospital discharge as particularly stressful, as leaving the hospital was experienced as a loss of supervision and feeling of safety that their HCPs provided them. One participant even expressed a strong wish to remain hospitalised for longer (Figure 2B). Furthermore, participants reported a perceived loss of independence compared with life before diagnosis, uncertainty about how living with HF would affect their way of life, and concern about the extent to which they would need to depend on others (Figure 2C).

When reflecting on the current monitoring process and their transition to life back at home, participants described an initial adjustment period during which they had to get used to incorporating monitoring into their daily lives. However, after this initial phase, they were able to integrate the measurements into their routines in a way that felt manageable, even though sometimes the requested timing of the measurement was unfortunate (Figures 2D–F). All participants were able to clearly explain the agreements they had made with the hospital regarding monitoring frequency and follow-up procedures, and they self-reported adherence to these agreements. A participant stated that their adherence was not solely based on obligation. They described monitoring as contributing to a sense of safety and of being heard. In addition, participants expressed a sense of shared responsibility with the HCPs toward their own health (Figure 2F). Participants further reported positive effects of participation in the RPM programme, including fewer hospital visits and increased reassurance regarding their condition (Figure 2G).

However, alongside these practical benefits, participants also highlighted an emotional dimension associated with RPM and living with HF. As being diagnosed with and living with HF already had a substantial impact on their daily lives, participants expressed a desire not to be reminded of their condition more often than necessary (Figure 2H). Especially participants in the stable phase of monitoring articulated a strong wish “to be able to go on with their lives”.

In this context, participants reflected on the importance of the unobtrusiveness of the RPM system (Figures 2I,J). They described that, by disappearing into the background of daily life, the system would allow them to more easily forget about its presence and thereby support a sense of normality. After an initial period of familiarisation, such unobtrusive monitoring was perceived to enable more honest everyday behaviour. According to multiple participants, constant awareness of being monitored could lead individuals to modify their behaviour or attempt to circumvent the system when not adhering to therapy. This dynamic was illustrated more explicitly in the first phase of the interview, when participants discussed a less serious, hypothetical “who would sit the least-match” with family members. In this example, more visible and tangible solutions, such as blankets placed on chairs and couches, were described as easy to avoid or move to win the sitting competition (Figures 2K,L). Similar behaviour was reported in the context of actual RPM use, with one patient mentioning on purpose reducing salt intake only on the day preceding scheduled measurements (Figure 2M). While reflecting on this behaviour during the interview, the same participant noted that this form of “cheating” nevertheless resulted in a healthier choice, leading them to consider whether some degree of system obtrusiveness might be beneficial after all (Figure 2N).

To provide patients with an initial sense of control, the proposed future RPM system included a manual confirmation button that required patients to actively transmit their monitored data to their HCPs. This design would allow patients to review their data before sending it, while the remainder of the monitoring process would occur automatically. However, participants' responses indicated that if the monitoring process were largely automated, they would prefer the final transmission step to be automated as well. Participants reflected that introducing an additional manual step would create unnecessary friction in the monitoring process, potentially leading to annoyance and reduced adherence if the action were perceived as superfluous, especially if the data was already measured automatically by the system (Figures 2O,P).

When patients are not actively interacting with the RPM system, while maintaining a sense of safety and continuous support, the RPM system should still allow them to carry out their usual activities unobtrusively. Participants expressed a desire for the monitoring to extend beyond the boundaries of their home, enabling the system to accompany them during their everyday activities. They also proposed the idea that the system extending outside of their house could allow for the system to also collect richer contextual information about their wellbeing, like activity data (Figures 2Q–S).

In conclusion, participants emphasised that future RPM should be unobtrusive, integrated into their daily lives, and adaptable to the various situations in which a person may find themselves. It should foster a sense of safety, enabling patients to feel seen and supported without becoming intrusive.

#### Data feedback

**Figure 3:**
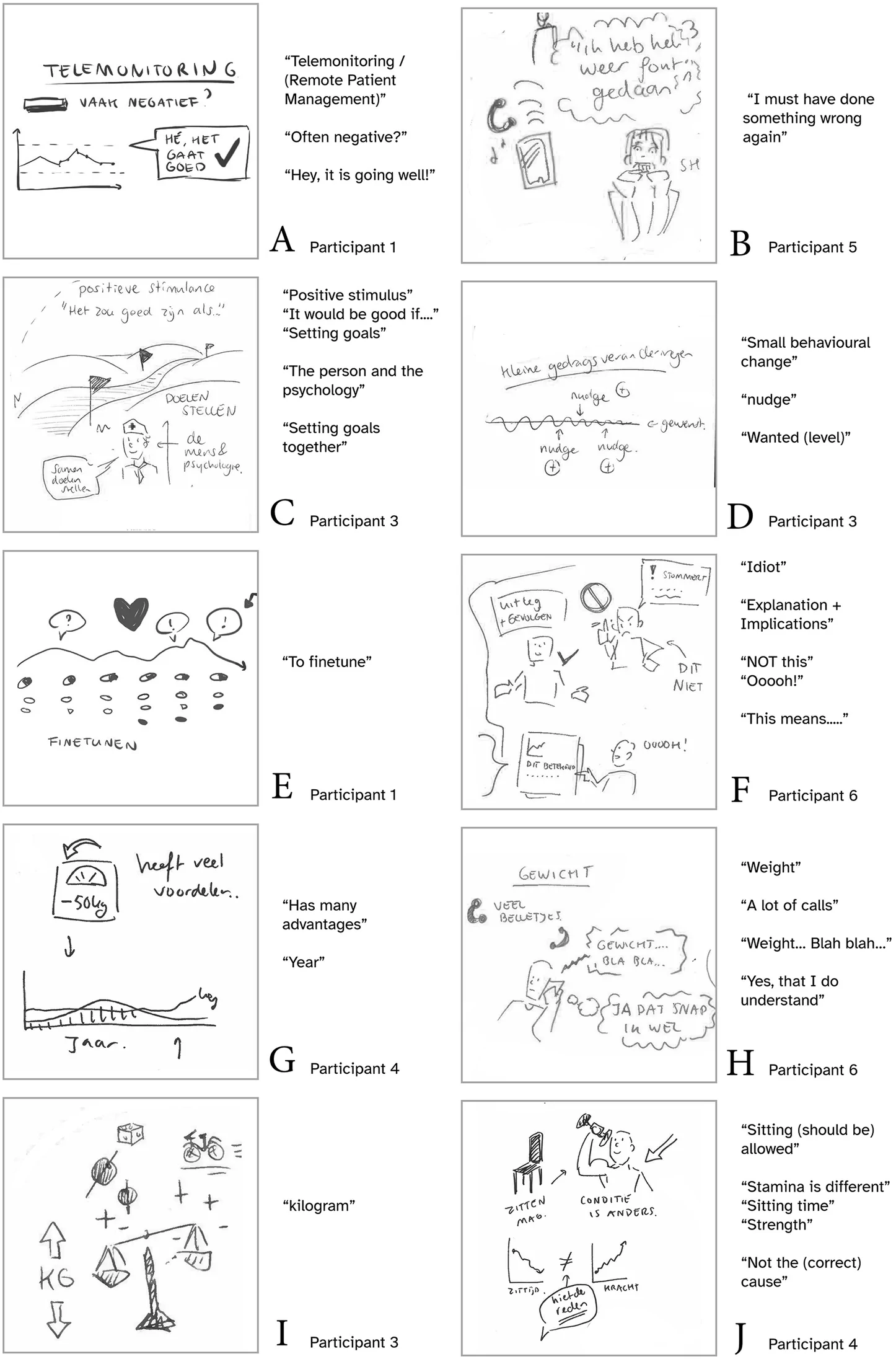
Researcher generated sketches, supplemented with participants' reflections shown in a different color. English translations are provided in the final column and are presented from top left to bottom right. Translations shown in brackets indicate additions made to improve clarity in English. **(A)** Researcher-generated sketch of an answer given by participant 4, depicting measured data by the participant. Feedback on this data is only given when it indicates a problem. The participant wishes for more positive feedback. **(B)** Researcher-generated sketch of an answer given by participant 5, depicting the participant's fear of receiving feedback, as it has a negative connotation. **(C)** Researcher-generated sketch of an answer given by participant 3, depicting the participant's desire to set goals for their care with their HCP. **(D)** Researcher-generated sketch of an answer given by participant 3, depicting a potential method of nudging a patient towards a healthier lifestyle based on their data. **(E)** Researcher-generated sketch of an answer given by participant 1, depicting a potential method of finetuning medication use using RPM feedback. **(F)** Researcher-generated sketch of an answer given by participant 6, depicting their preferences regarding communication of monitored data. **(G)** Researcher-generated sketch of an answer given by participant 4, depicting their wish to see their data as trends over time to see progression. **(H)** Researcher-generated sketch of an answer given by participant 6, depicting their annoyance over receiving a lot of calls, or about information they understand. **(I)** Researcher-generated sketch of an answer given by participant 3, depicting the participant's confusion regarding changes in weight, and corresponding feedback. **(J)** Researcher-generated sketch of an answer given by participant 4, depicting the participant's want to measure, communicate and receive feedback on data that is directly relevant to their disease, using the warm-up scenario as an example.

Participants mentioned that in current RPM implementations, feedback is typically provided only when measurements fall outside predefined healthy ranges. As a result, contact from HCPs is often perceived as a signal of a potential problem, giving feedback a predominantly negative connotation (Figures 3A,B). Furthermore, participants expressed that especially during longer periods of good health, monitoring could feel demotivating due to getting no feedback. Participants expressed a desire for more positive and proactive feedback, like small, constructive nudges that could help them maintain or even improve their health status, personalised on their collected data and medication use (Figures 3C–E). This would give the participants' a greater feeling of control over their health, let them take more responsibility over their own health, and offer insight into positive health progression. These nudges didn't need to be intrusive like current actual alerts are, but something for the participants to check out in their own time, at their own volition.

To make the feedback actionable for participants, the following requirements regarding the presentation of the data emerged during the conversations: It should be kind in its communication, to foster a non-judgemental environment; educational, to not just tell them something is wrong, but also foster understanding about their condition; and digestible, allowing insightful interaction with the data in a short amount of time (Figure 3F). One Participant likened it to reading a quick news article while their coffee was brewing. Furthermore, participants appreciated the idea of visualising their data as trends over time, as this would enable them to see their progression (Figure 3G). In the participants eyes, allowing this could not only foster understanding, but also create a feeling of self-efficacy and control of their own wellbeing, standing opposed to the mentioned fear of loss of independence.

Participants further described situations in which measurements they personally interpreted as a positive trend triggered negative alerts, leading to confusion. One example concerned weight loss, which participants often perceived as a positive change, while within the RPM system it could trigger an alert to the hospital as a potential sign of HF deterioration. Participants reported feeling annoyed in such situations, especially when the weight loss was a perceived result from adherence to prescribed therapy or dietary instructions (Figures 3H,I).

Participants emphasised the importance of measuring values that reflected what HCPs aimed to assess, as well as the need for clear explanations of why alerts were generated. During the introductory phase of the interviews, when monitoring was discussed using the example of a sitting competition, a participants highlighted the difference between measuring sitting time and measuring actual physical activity, where they deemed the latter more logical and easier to understand why it needed to be measured (Figure 3J).

Thus, participants expressed a desire for RPM feedback that is reassuring and positive, concise and clear with actionable guidance and presented in a way that fosters understanding, engagement, and a sense of control over their health.

#### Invasiveness

**Figure 4:**
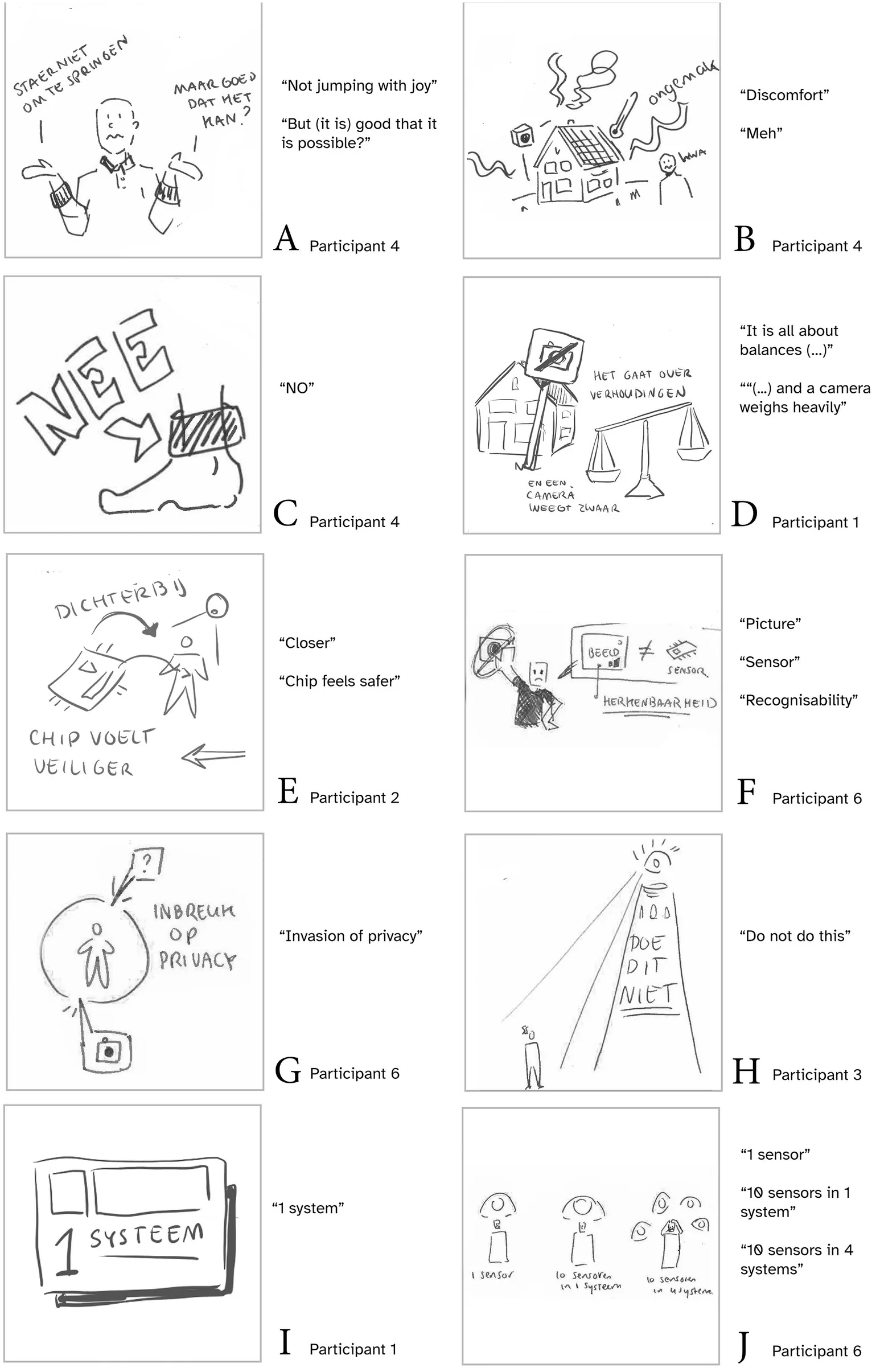

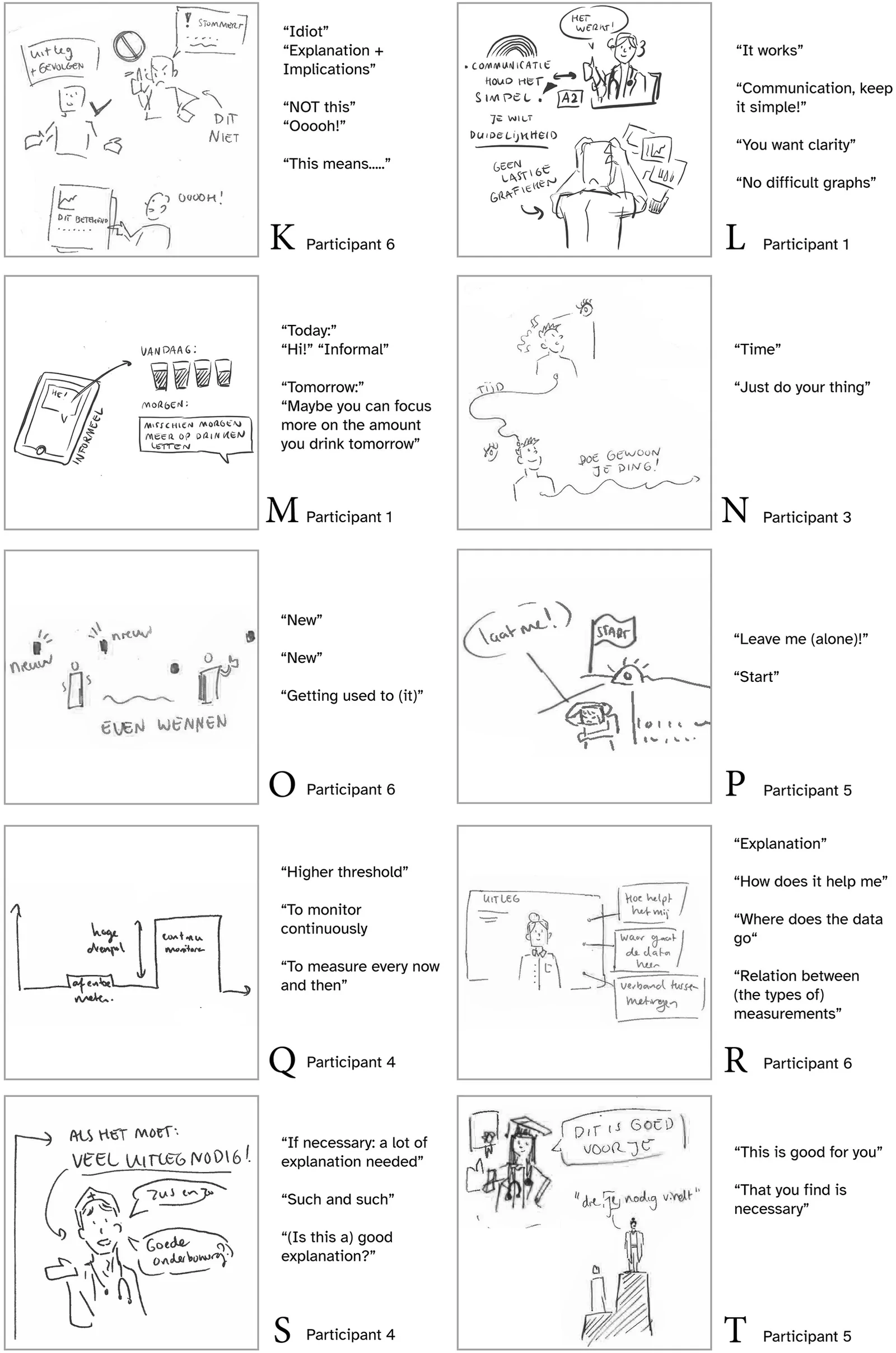
Researcher generated sketches, supplemented with participants' reflections shown in a different colour. English translations are provided in the final column and are presented from top left to bottom right. Translations shown in brackets indicate additions made to improve clarity in English. **(A)** Researcher-generated sketch of an answer given by participant 4, depicting their doubt regarding multisensory monitoring, while also seeing that there is potential for the technology. **(B)** Researcher-generated sketch of an answer given by participant 4, depicting the discomfort a house full of sensors could potentially give the participant. **(C)** Researcher-generated sketch of an answer given by participant 4, depicting an ankle bracelet, which the participant used as a metaphor to convey a feeling of being shackled by the monitoring system. **(D)** Researcher-generated sketch of an answer given by participant 1, depicting the participant's hesitancy to camera-based monitoring due to the perceived intrusiveness of such a system. **(E)** Researcher-generated sketch of an answer given by participant 2, depicting that the participant would rather be monitored using an implanted chip, instead of a camera. **(F)** Researcher-generated sketch of an answer given by participant 6, depicting how an implanted chip forgoes the recognizability of the participant. **(G)** Researcher-generated sketch of an answer given by participant 6, depicting the participant's fear of camera's breaching their privacy. **(H)** Researcher-generated sketch of an answer given by participant 3, depicting the patient wishes to not continuously be watched by the system. **(I)** Researcher-generated sketch of an answer given by participant 1, depicting their wish for the RPM system to be a single system. **(J)** Researcher-generated sketch of an answer given by participant 6, depicting their preference regarding the set-up of monitoring systems. **(K)** Researcher-generated sketch of an answer given by participant 6, depicting their preferences regarding communication of monitored data. **(L)** Researcher-generated sketch of an answer given by participant 1, depicting the participant's wishes for easy communication of monitored data. **(M)** Researcher-generated sketch of an answer given by participant 1, depicting their wish of communication of monitored data to be the form of informal messages. **(N)** Researcher-generated sketch of an answer given by participant 3, depicting them getting used to the system after some time of use. **(O)** Researcher-generated sketch of an answer given by participant 6, depicting them getting used to the system after some time of use. **(P)** Researcher-generated sketch of an answer given by participant 5, depicting them having more issues with being monitored at the start of their monitoring. **(Q)** Researcher-generated sketch of an answer given by participant 4, depicting the participant's view on how more intrusive monitoring techniques would require larger effort to get used to. **(R)** Researcher-generated sketch of an answer given by participant 6, depicting their wish for their HCP to explain RPM well. **(S)** Researcher-generated sketch of an answer given by participant 4, depicting their need for proper explanation if more obtrusive systems would be introduced in their homes. **(T)** Researcher-generated sketch of an answer given by participant 5, depicting the participant's view of their healthcare provider.

Participants reported that imagining the described future monitoring involving multiple sensors elicited discomfort, causing them to be hesitant about the adoption of such a system. This was mainly due to perceptions of increased invasiveness compared to the current implementation (Figure 4A). They emphasised that this level of perceived invasiveness depended on how the technology was implemented in their homes. Poorly integrated systems were considered to risk disrupting the home as a place of comfort and privacy by creating a continuous sense of discomfort (Figure 4B). One participant even described the envisioned experience as having to wear a prison anklet monitor, using it as a metaphor to convey a feeling of being mentally shackled by the system to their condition (Figure 4C). While discussing the potential future monitoring system, several considerations emerged that participants believed would influence how the invasiveness such systems might be perceived.

There was strong resistance to camera-based monitoring. Participants even stated a preference for a more physically invasive implanted chip to measure vital signs over being monitored via camera (Figures 4D–F) Cameras were perceived as overreaching by potentially capturing private moments of their life that participants felt were unrelated to their medical condition (Figure 4G). In addition, participants anticipated that camera-based monitoring could create a persistent sense of being watched, thereby reinforcing an unwanted awareness of both the monitoring itself and their role as a patient (Figure 4H).

Fragmented systems, with multiple devices spread throughout the home, were perceived as more invasive, as they were both visually obtrusive and they extended the sense of being monitored to more parts of the house. Participants expressed a preference for solutions integrated into a single device, which would reduce intrusion and limit awareness of the system occupying their personal space (Figures 4I,J).

Participants emphasised that feelings of invasiveness would not arise solely from being monitored, but also the systems communicative abilities (Figure 4K). Complex or unexplained visualisations, such as graphs, could create the impression of insufficient understanding of their own condition, amplifying uncertainty and the sense of being judged (Figure 4L). Instead, participants expressed a preference for a slightly informal, conversational tone in the system's communication, as they felt this would make the interaction more inviting and supportive rather than just evaluative (Figure 4M).

Participants noted that perceived invasiveness was most prominent immediately after the system was introduced and typically diminished over time as they became accustomed to it (Figures 4N–P). Systems that caused larger deviations from participants' normal routines were expected to feel more invasive (Figure 4Q). Several participants indicated that more invasive monitoring could be acceptable if HCPs clearly explained why it was necessary for their safety, emphasising the importance of trust, transparency, and justification (Figures 4R,S). Many participants reported inherent trust in their HCPs, believing they would act in the patient's best interests (Figure 4T). One participant also mentioned that the intensity of monitoring should correspond to disease severity, with more intrusive systems being acceptable for conditions requiring closer clinical oversight (Figure 4D).

Thus, while more elaborate monitoring has the potential to be perceived as more invasive in the participants life, there are several design and systemic considerations that can help alleviate this problem.

#### Contact with healthcare providers

Participants reported that in the current RPM system when one or more metrics fall outside predefined thresholds, HCPs are alerted. After reviewing the data, they may choose to contact the patient by phone to assess whether any issues are present and whether medical adjustment is required.

**Figure 5:**
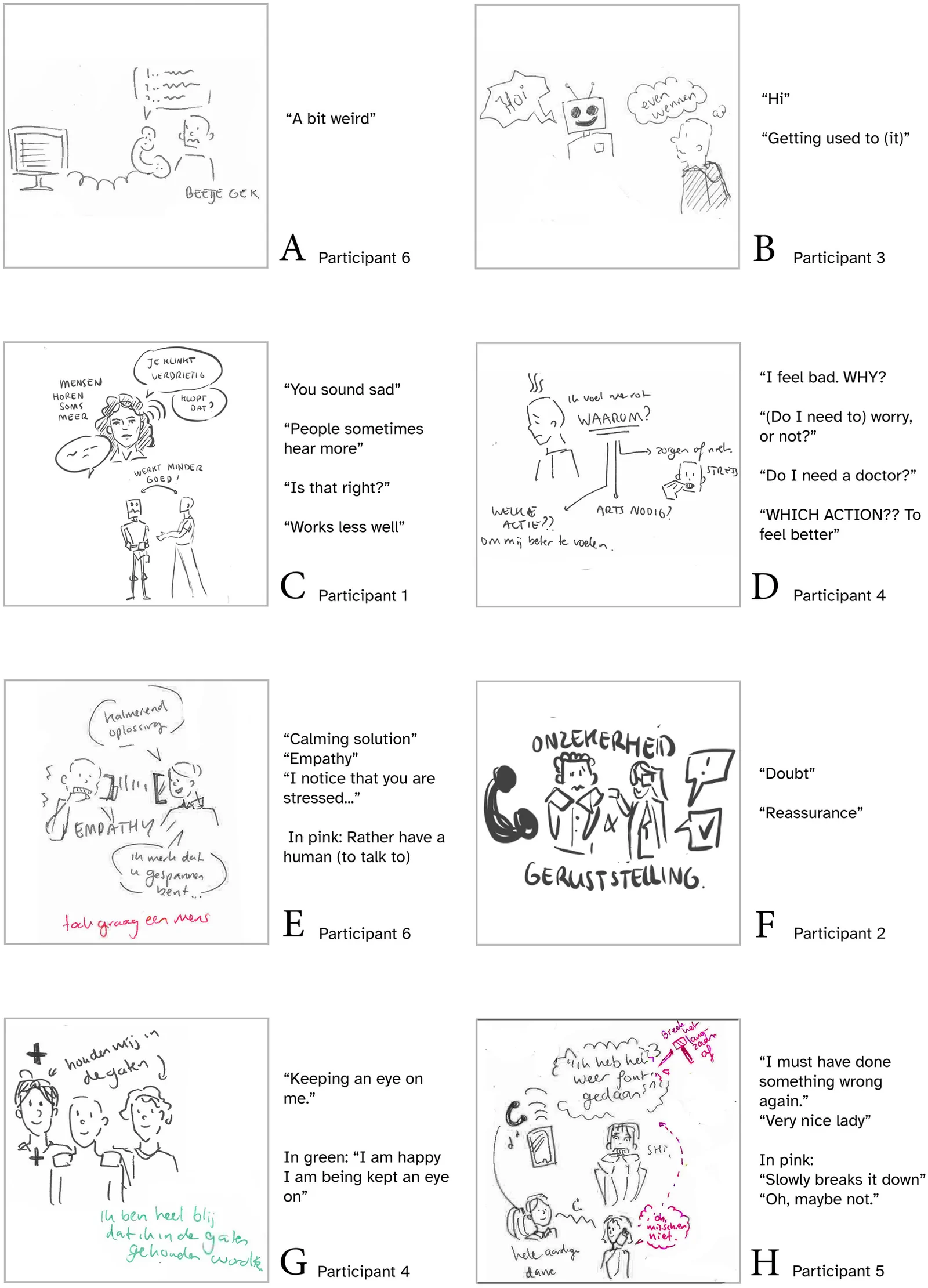
Researcher generated sketches, supplemented with participants' reflections shown in a different colour. English translations are provided in the final column and are presented from top left to bottom right. Translations shown in brackets indicate additions made to improve clarity in English. **(A)** Researcher-generated sketch of an answer given by participant 6, depicting their uncomfortable reaction to calling the hospital and receiving an automated e-nurse. **(B)** Researcher-generated sketch of an answer given by participant 3, depicting them needing to get used to interacting with an automated system instead of a human HCP. **(C)** Researcher-generated sketch of an answer given by participant 1, depicting the differences between human and automated interaction, with the human interaction being perceived as being able to interpret emotions better. **(D)** Researcher-generated sketch of an answer given by participant 4, depicting the types of information the HCP provides to the participant to help them feel better on days they feel bad. **(E)** Researcher-generated sketch of an answer given by participant 6, depicting an HCP showing personal empathy towards the participant. **(F)** Researcher-generated sketch of an answer given by participant 2, depicting the participant getting reassurance from their human HCP. **(G)** Researcher-generated sketch of an answer given by participant 4, depicting the patient being comfortable in being kept an eye on by their HCPs. **(H)** Researcher-generated sketch of an answer given by participant 5, depicting the way HCPs break through the self-doubt that the participant harbored.

When presented with the idea of a system in which feedback from the main HCP could be omitted or replaced by an automated e-nurse, all participants expressed hesitation, repeatedly emphasising the importance of human contact (Figures 5A,B). Participants valued the understanding shown by their HCPs and appreciated the genuine interest they displayed in them as people and their circumstances (Figure 5C). The calls with the HCPs were experienced as reassuring and humanizing, with extra attention towards the context of the measurements, instead of just the values. This was especially valued on days when patients were not feeling well, even when the measurements did not indicate any irregularities, as the HCP was able to support them in identifying actions to feel better (Figure 5D). Furthermore, participants reported that nurses remembered them from earlier interactions, which created a sense of familiarity and personal connection (Figures 5E–G). The previously mentioned fear of judgement was reported to decrease during these interactions, as HCPs helped ease personal doubts and offered actionable feedback that allowed participants to regain a sense of control (Figure 5H). Through these supportive and reassuring interactions, HCPs helped alleviate patients' doubts, supported them in interpreting their symptoms, and encouraged confidence in managing their condition by guiding appropriate self-management actions or, when necessary, confirming the need for additional care.

## Discussion

This study explored patient expectations for future RPM in patients with HF by employing a participatory design methodology, co-constructing stories. By narrating and envisioning a future scenario of life with RPM, patients were invited to reflect on their current experiences and extend these into the envisioned future. This approach generated insights into the characteristics and features patients with HF consider important for future RPM.

### Experiences with current RPM

Overall, participants reported a positive experience with current RPM. They described a range of benefits, including greater reassurance about their health, feeling seen by healthcare professionals, and fewer hospital visits. This way, the interviews revealed that RPM is not just a clinical tool, but that it also plays an important supportive role in regaining confidence and control, particularly for those who struggled to adjust to life with HF, something which is commonly reported among patients (27–30). These findings are consistent with previous and recent studies, in which participants similarly reported increased feelings of security. These were partly attributed to feeling more closely connected to their healthcare professionals, a pattern that was also reflected in the experiences described by participants in the present study (30–35).

Participants noted several drawbacks of current RPM. Contact often occurred only when measurements suggested potential problems, causing some participants to associate RPM with negative health developments, an experience reported in earlier research (30, 35). Integrating monitoring into daily routines was challenging, and discrepancies between patients' perceptions of their health and the generated alerts by the system led to confusion and reduced confidence. These issues highlight the need for clear communication and understandable monitoring processes to minimise uncertainty, a finding that is also reflected in other qualitative studies on patient experiences with RPM (28, 35).

### Characteristics of co-constructed future RPM

Taking these experiences into account, and after being presented with a narrative describing potential future developments in RPM, participants reflected on their expectations and preferences for future RPM. A central theme was the desire for unobtrusive systems. While participants valued the sense of safety and recognition that monitoring could provide, they emphasised that they preferred to not be continually be reminded of being monitored and their chronic disease. These preferences align with findings from previous qualitative studies, which suggest that the repetitive nature of RPM can become burdensome for patients (36, 37). Goevaerts et al. describe that most dropouts in their study were caused due to more obtrusive characteristics of RPM, such as the patient having to do manual measurements, the system having technical difficulties and low usability. Some of their participants also reported discontinuing monitoring due to the mental stress it caused by making the participants focussed on their illness (35, 37). Relatedly, in a qualitative interview study by Micheluzzi et al. exploring heart failure patients' experiences with continuous monitoring, patients reported feeling tied to their monitoring technology. They described a perceived inability to spend time away from home, as doing so could prevent them from transmitting data to their healthcare professionals and created concerns about potential issues occurring while they were away (30). To avoid causing these issues, RPM systems should have high usability and be able to seamlessly integrate into daily life, regardless of location and activity.

Participants further stressed that the feedback based on measured data should be actionable, clear, and constructive. They expressed a preference for feedback that not only informed them of their status but also supported them in making behavioural adjustments and taught them self-management. In this way, RPM could contribute to them learning about their condition. Patients who are confident in self-management tend to be more comfortable with RPM, suggesting that fostering this confidence may increase their comfort with remote care (28, 38). In addition, higher self-management confidence is associated with better long-term treatment adherence and aligns with literature showing that educational components embedded within RPM systems can improve clinical outcomes (4, 39). To foster patient self-management, it may be beneficial for HCPs to discuss patient expectations regarding RPM at the outset and jointly develop an actionable digital care plan. Such a plan can then be used not only to provide feedback when signs of decompensation occur, but also to deliver positive nudges that support patients in their self-management while aligning with their preferred level of engagement with the monitoring. A similar approach was performed in a study by Østrem et al., in which an RPM programme combined with nurse calls providing tailored feedback was employed to help patients identify symptoms and improve lifestyle, resulting in continued self-monitoring and lifestyle changes even after patients were phased out of the programme (32).

Participants acknowledged that more elaborate monitoring has the potential to feel intrusive, but they also highlighted several design considerations that could mitigate these concerns. Key recommendations included keeping monitoring contained to a single location in the home, providing clear and supportive communication about the purpose of monitoring, and aligning the intensity of monitoring with patient needs and clinical context. Participants emphasised that healthcare professionals should only monitor metrics that meaningfully contribute to clinical decision making. In case more invasive monitoring would become necessary for a patient, a clear explanation by their HCP at the start of the RPM program about the reasoning behind the monitoring intensity increase trust in the HCP and RPM program, increase patient knowledge about their condition and improve effectiveness of RPM (40). Nonetheless, participants reported a strong aversion to camera-based monitoring, which was perceived as moving beyond the collection of physiological data towards surveillance. This finding aligns with earlier literature on video-based medical monitoring, in which even a promised 20% increase in the detection of worsening disease was insufficient to convince patients to place a camera in their home. This was mainly due to the recognisability of the patient, and distrust that the camera was only collecting data for healthcare related purposes (36).

Factors like the HCP's interpersonal skills and communication style are often related to patient trust in their HCP and their delivered care (41). In line with this, proposing to remove direct contact with HCPs, and replacing it with automatic alerts and advice was therefore not a liked option. Participants expressed a clear preference for maintaining meaningful human contact and valued healthcare professionals' ability to engage with them. HCPs have a lot of effect on how patients experience care, and by extend RPM. They can provide context, reassurance, and humanity to a situation that is inherently stressful (33–35, 40, 41). These findings suggest that while RPM may change how care is delivered, the presence and engagement of healthcare professionals remain essential to patients' experience of care, highlighting the value of a socio-technical system in which RPM technology supports and strengthens the role of HCPs, instead of replacing them.

Trust is considered a primary factor in a patient-HCP relationship, not only in face-to-face care but also in remote care (42). Without trust between the patient and HCP, patients may withhold information or avoid seeking care. This behaviour may reflect attempts to protect themselves from potential stigma or from intrusion into their personal lives (43). In the context of RPM, trust should not only be considered within the patient–HCP relationship, but also in terms of patients' trust in their own capabilities and in the RPM system itself, as a lack of trust may lead to non-use of RPM. The RPM characteristics identified by participants appear to support the (re)building of such trust, for example through clear communication with HCPs, the provision of actionable data, and non-intrusive system design. Furthermore, it allows for a reported feeling of continuity of care, when patient believe that clinical decisions are made based on their data (30). These aspects should therefore be carefully considered when designing and implementing future RPM interventions.

### Co-constructing stories and sketching

The co-constructing stories approach provided a structured way to create deeper understanding of participants' situations and perspectives. By initially prompting the participants with a prospective narrative design probe and letting them slowly alter it to better fit their ideal situation of future RPM, we elicited discussion in which requirements and wishes for future RPM emerged. By being encouraged to situate RPM in their daily lives, participants were encouraged to not only consider moments of direct interaction with the monitoring system, but also how its presence might shape other aspects of their daily lives. By walking through a typical day in detail, participants reflected on how RPM could influence small, everyday moments, rather than focusing solely on the act of monitoring. Introducing alternative scenarios, such as days when family members were visiting or when participants felt unwell, further enriched these reflections. These variations prompted participants to reconsider earlier responses and situate monitoring within a broader range of lived circumstances, steering the final requirements towards a system suitable for day-to-day life, instead of just system use.

In this study, the two interviewers also analysed the sketches. An alternative approach would be to involve a third, independent researcher to apply the AVA method to the researcher-generated sketches and explore whether similar themes and underlying messages emerge compared with those identified by the researchers who were present during the interviews. To facilitate this process, the independent researcher should have access to both the sketches and recordings of the sessions (either audio or video). This approach could provide an additional perspective on the interpretation of the visual data and on how visual representations contribute to identifying participants' needs and perspectives.

### Limitations

Six patients with HF participated in this interview study. Although all participants had experience with RPM, their perceptions may also have been influenced by broader experiences with the healthcare system, as negative healthcare experiences can shape how patients perceive later care encounters (44). During the interviews, two participants referred to earlier negative experiences related to other conditions, which may have shaped their views on RPM. Clarifying questions and sketch notes were used during the interviews to encourage participants to reflect specifically on the monitoring experience.

One finding may also relate to the stage of HF at which participants engaged with RPM. All but one participant was in the stable phase of monitoring, meaning they were considered clinically stable and may experience fewer or milder symptoms than recently admitted patients (6). Patients in this stage may be more focused on resuming daily life, which could influence their preferences regarding the intensity of monitoring. In contrast, patients in a less stable phase might value more frequent or rigorous monitoring due to greater feelings of vulnerability (45). Even so, all participants had experienced this unstable phase of living with HF and being monitored more rigorously.

This study focused specifically on patients with HF, which should be considered when interpreting the applicability of the findings to other chronic disease populations. As RPM is increasingly used in conditions with different levels of disease burdens, such as diabetes and asthma, experiences related to uncertainty in living with the condition, monitoring intensity, and intrusiveness may differ across patient groups. Nevertheless, several themes identified in this study, including the importance of HCP contact, privacy within the home environment, and clear, understandable communication of health data, are likely relevant beyond HF care alone. Future research should therefore examine whether these experiences are consistent across patients with different chronic conditions.

Another limitation relates to the age of this study's participants The average age of participants was lower than the typical age of patients with HF (46). With RPM involving the use of digital technology, age-related differences in comfort with such technologies may have influenced participants' expectations. This limitation is further exacerbated by the fact that patients included in this study were already deemed by their HCPs to have sufficient digital literacy to participate in RPM and did not discontinue using RPM. Consequently, the views captured in this study may not fully reflect those of older, or less digitally literate, patients with HF, who may encounter different challenges or preferences with regards to RPM.

Finally, the sketcher was involved in both generating the visual representations during the interviews and analysing these sketches to derive the results. This dual role may have introduced an element of early researcher interpretation during data generation. To mitigate this potential influence, participants were encouraged to actively engage with the sketches by reflecting on and modifying them as they were created and were periodically invited during the interview to validate the emerging visual interpretations.

## Conclusion

A co-constructing stories methodology was used to explore the wishes and expectations of patients with HF regarding future RPM, yielding valuable insights into patient perspectives. Patients emphasised that RPM should be providing actionable feedback, be minimally invasive, adaptable to daily life, and complemented by meaningful human contact. Participants preferred RPM systems that were minimally disruptive to daily life and capable of fading into the background, while still supporting active self-management through appropriate guidance and actionable feedback. Through the co-constructing stories method, the results of this study revealed motivations and latent needs of patients with HF, providing insights for designing patient-facing components for remote monitoring and care.

## Data Availability

The datasets presented in this article are not readily available due to the personal nature of the data used in this study, the study's data will be made available only upon reasonable request to the corresponding author. Requests to access the datasets should be directed to Wessel Nieuwenhuys, w.w.nieuwenhuys@tue.nl.
