# Supplementary material for: Co-constructing stories with patients with heart failure to envision future remote care possibilities: a qualitative interview and sketching study
Source: Front Digit Health. 2026 Sep 3;8:1894748. doi: 10.3389/fdgth.2026.1894748 (PMC13582375; doi:10.3389/fdgth.2026.1894748)
Supplement: Supplementary file 1 [file Datasheet1.docx]

# Appendix A: Translated Prompts and Interview Questions

**Section 1: Practice Scenario**

You have a bet with your family. Everyone claims to sit the least during the weekends. You aren't quite so sure about that.

That is why you have come up with a challenge: you are going to keep track of how often you sit down and stand up during the day, and how much time you spend sitting.

This is very easy, because there is a product specifically made for monitoring sitting time: the sitting blanket! You simply throw this blanket over your couch or chair, and it keeps track of how long you are lounging on the couch!

Now, the whole family is participating, and you are going to track your sitting habits for 4 weeks.

Imagine this: It is day 1 of the challenge, a quiet Saturday morning, and the blankets are on your couch and chairs.

***Guiding questions:***

- *What would your experience be like? How do you think and feel about it? What does your day look like?*
- *Would you behave differently in this situation?*
- *If so, how and why? How do you feel about this change in behavior?*
- *What would your desired behavior be? And what’s your corresponding desired feeling?*

**Section 2: Semi-structured Interview Current RPM**

Currently, you are using RPM. You are now doing this via a mobile app, in which you enter your values yourself. Namely blood pressure, weight, resting heart rate, and a questionnaire (Symptoms: Shortness of Breath, Fatigue, Swallowing, Dizziness, Palpitations)

- What are your current experiences with RPM? How do you feel before, during, and after interacting with the RPM system?
- What does your average day look like now?
- What is your current living situation?
- How independent do you feel from your partner, friends, and/or family? How do you feel, for example, when they are not available?
- Are there things in day-to-day situations that you struggle with?
- How do you explain the RPM system to friends/family?
- (How) does remote monitoring fit into your daily life?

**Section 3: Main Scenario**

We have developed a device, called VITALL, that automatically tracks the following health indicators: heart rate, physical activity at home, body temperature, fluid intake, medication intake, and the sleep-wake cycle. VITALL does this automatically and continuously.

The only thing you still need to do is weigh yourself in the morning. Furthermore, you have the option to view your information at any time of the day or to add your experiences. At the end of the day, you send the data to the doctor yourself.

Now imagine having this device at home.

***Guiding questions:***

- *What would your experience be like? How do you think and feel about it? What does your day look like?*
- *Would you behave differently in this situation?*
- *If so, how and why? How do you feel about this change in behavior?*
- *What would your desired behavior be? And what’s your corresponding desired feeling?*
